# Potential Use and Challenges of Constructed Wetlands for Wastewater Treatment and Conservation in Game Lodges and Resorts in Kenya

**DOI:** 10.1155/2020/9184192

**Published:** 2020-10-14

**Authors:** Richard O. B. Makopondo, Laban K. Rotich, Cynthia G. Kamau

**Affiliations:** ^1^The Technical University of Kenya, School of Hospitality and Human Ecology, Department of Hospitality and Leisure Studies, P.O. Box 52428-002000, Nairobi, Kenya; ^2^Conservation and Environmental Management Consultant, NEMA Associate, P.O. Box 6431-00200, Nairobi, Kenya

## Abstract

Constructed wetlands are cost-effective wastewater treatment alternatives that receive worldwide acceptance. For the Kenyan hospitality industry, in particular, constructed wetlands (CWs) provide opportunities for wastewater reuse and recovery of resources, as well as improvements in local environmental conditions. Hospitality establishments produce large volumes of wastewater that is sometimes discharged to the environment without being treated. This is not only harmful to communities living downstream of these rivers but also to the flora and fauna which are the main attraction for most who visit these lodges. This study used qualitative methods to collect and analyze published official government documents, peer-reviewed research articles, and professional reports including leading international and Kenyan case studies to explore how constructed wetlands can be effectively used in game lodges and resorts situated in arid and remote areas of Kenya. The study investigated wastewater management systems adopted by remote game lodges and resorts in Kenya and the potential role, as well as the challenges to adopting constructed wetland (CW) technology. The results indicated that hotels, game lodges, and resorts both internationally and locally are adopting different types of CWs including surface and subsurface flow as alternative nature-oriented wastewater management systems. The study identified opportunities in the use of CWs as a wastewater management and conservation strategy. The results suggest that there are potential challenges which include inadequate expertise and technical support; low volume of discharge during off-seasons; limited space or land; and the attitude of hospitality managers towards constructed wetlands. Based on these preliminary findings, one may conclude that game lodges, ecolodges, and resorts in remote areas are prime candidates for constructed wetland establishment. The study makes specific recommendations with implications for policy and practice to promote sustainable hospitality operations and environmental conservation. It is suggested that future studies test the efficacy and efficiency of CW technology as wastewater management systems in the Kenyan wilderness areas including national parks, game reserves, and forests.

## 1. Introduction

In many developing countries including Kenya, inadequate, uncoordinated policies and measures on waste and wastewater management coupled with weak legislation, inadequate standards, and lack of monitoring and regulations have led to ineffective wastewater treatment [1]. As a result, loss of water quality has played a major role in a number of environmental issues, including the availability of human drinking water and the survival of biodiversity. Today, some 1.1 billion people have no access to clean drinking water, and 2.6 billion have no adequate sanitation [2]. In general, due to human economic activity, the quality and quantity of environmental resources are increasingly being impacted.

In line with calls for sustainable tourism growth and increased environmental awareness among tourists, government agencies, and associations, environmental management has become a critical issue for hotels [3]. Pressure on the environment of tourism destinations has increased and sometimes with adverse impacts, despite the apparent positive impacts of hotels. Tourism and hospitality industries consume natural resources such as water and solid fuel from the environment that form part of the production resources so as to produce final products and services to tourists. Additionally, a esthetic value of the environment is one of the key attractions for visitors. However, hospitality and tourism operations contribute to large amounts of solid and liquid wastes, which can be deposited to rivers and other unapproved locations and which, if not properly controlled, may lead to negative ecological disruptions [4], contributing to increased water contamination, waste, and water depletion, which jeopardize water access and thus supplement the safety of the river [5]. These challenges as well as potential negative impacts point to the need for wastewater management and environmental conservation strategies to ensure integrity of the local environment.

Effective wastewater management is a continuing challenge for Kenyan households, hotels, and industries. Hotels generate a great deal of solid and liquid waste through their operations and are occasionally discharged into water systems and other unapproved sites [4]. Sanitation in rural areas, especially in developing countries such as Kenya, is characterized by inadequate investments for centralized water supply systems that are often unaffordable given the remote locations and lack of financial resources [6], for instance, the arid remote areas in which the Kenyan game lodges are situated area of great natural and economic value, which are visited by a considerable number of tourists each year. Most hotels in these regions are not treating wastewater correctly but are using soak-away pits leading to contamination of ground water and rivers (https://youtu-be/D3phkRaHZ-Q).

While some establishments have policies on wastewater management, the extent to which government policies on wastewater discharge are implemented or successful in realizing desired environmental conservation goals is not clear. There is a need for simple yet sustainable solution to wastewater management that is nature-oriented. It is imperative that the industry adopts technologically affordable yet environmentally-friendly approaches towards wastewater disposal [7].

Therefore, this study sought to explore the adoption of constructed wetlands by hospitality establishments and their potential role, as well as the challenges to their adoption as wastewater management systems in game lodges and resorts in the arid and remote areas in Kenya. The specific objectives were (i) to explore wastewater management systems adopted by hospitality establishments globally as well as remote game lodges and resorts in Kenya; (ii) to identify the opportunities and challenges to adopting constructed wetland technology in remote game lodges and resorts in Kenya; and (iii) to investigate the efficacy of constructed wetlands as wastewater management systems in remote game lodges and resorts in Kenya.

Hospitality and tourism ventures are faced with increasing amounts of wastewater during peak seasons and costs associated with the management of wastewater. Most hospitality facilities generate large massive amounts of solid and liquid waste which, if not properly managed, can lead to negative environmental, disease, and a esthetic effects. Popular wastewater sources include showers, baths, wash-up cups, and wash-ups, including kitchen sinks and automatic wash-ups with high organic material levels which promote bacterial growth.

## 2. Wastewater Management in Hotels and Resorts

Several hotels take environmental problems seriously and start activities such as recycling, conservation of water and energy, environmental education, afforestation (tree planting), and waste and water management. Wastewater projects for hotels and resorts require highly effective wastewater treatment systems that can start up quickly, handle highly variable flows, operate quietly, produce no noticeable odor, and blend into landscaping. These systems range from basic low-cost wastewater reuse tools to direct toilets and outdoor landscaping to sophisticated sedimentation tanks, bioreactors, filters, pumps, and disinfection processes [8]. These systems vary in cost and energy requirements, usually with higher levels of treatment.

## 3. Constructed Wetlands as Wastewater Management Systems

To address the challenges associated with sanitation and wastewater management in nature-based tourism areas, hospitality establishments may use one of the three main categories of systems. The first is diversion systems, which do not store wastewater (but may filter and disinfect it) before immediate reuse. The second is the physical wastewater treatment system, which allows wastewater to be stored and treated with filtration and disinfection processes. The third system for treating biological wastewater includes BCWs which utilize the technology and approaches of biological wastewater processing.

Constructed wetlands (CWs) can be defined as man-made, engineered systems designed and constructed to use wetland vegetation, soils, and related microbial assembly and natural processes to support the treatment of wastewater by optimizing the physical, chemical, and biological processes that occur in natural wetland ecosystems [9]. El-Khateeb and El-Gohary [10] asserted that constructed wetlands are artificial wastewater treatment systems which comprise shallow ponds planted with aquatic plants. Constructed wetlands are effective wastewater treatment mechanisms that are ideal for developing countries for they involve minimal operational costs as well as simple technology [9]. CWs can be classified according to different parameters, but the two most important criteria are water flow regime (surface and subsurface) and the macrophytic growth. The surface flow, subsurface flow, horizontal flow, and vertical and hybrid systems comprise various forms of CWs.

## 4. Surface Flow Systems

**Figure 1:**
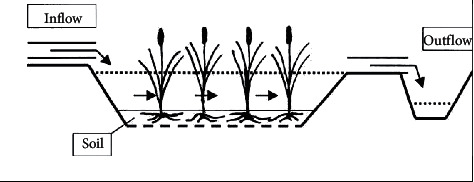
Schematic representation of the free-water surface constructed wetland with emergent macrophytes (source: [11]).

A typical surface flow (SF) or free-water surface constructed wetland (FWS CW) is shown in Figure 1. Schematic representation of the free-water surface constructed wetland with emergent macrophytes with emergent macrophysics (usually covering more than 50 percent) is a shallow sealed basin or sequence of basins, containing 20–30 cm of rooting soil with a depth of 20–40 cm of the emerging macrophytes (normally more than 50%). As the name suggests, wastewater flows across the atmospheric soil. Injection water containing particulate pollutants and dissolved pollutants depletes and slows down an area of low water and emerging vegetation [12].

Long retention times and a substantial surface area touching base with running water deliver an effective separation of particulate matter and organic matter. The sediment, plant biomass and plant litter surfaces are the areas where most of the microbial activity affecting water quality occurs as well as oxidation of organic matter and transformation of nutrients. Biomass decay provides a carbon source for denitrification, but the same decay competes with nitrification for oxygen supply [12].

## 5. Subsurface Systems

**Figure 2:**
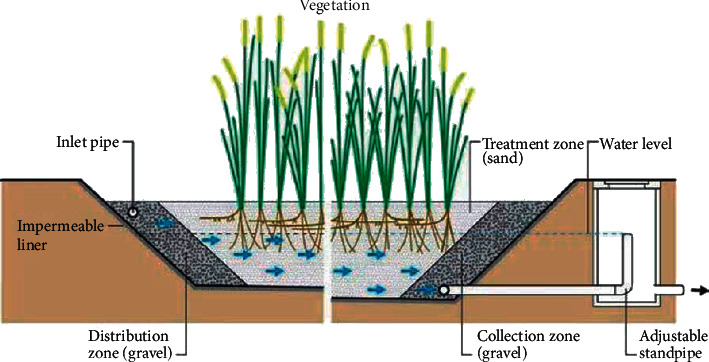
Schematic cross section of a horizontal-flow constructed wetland (HFCW) (source: [13]).

**Figure 3:**
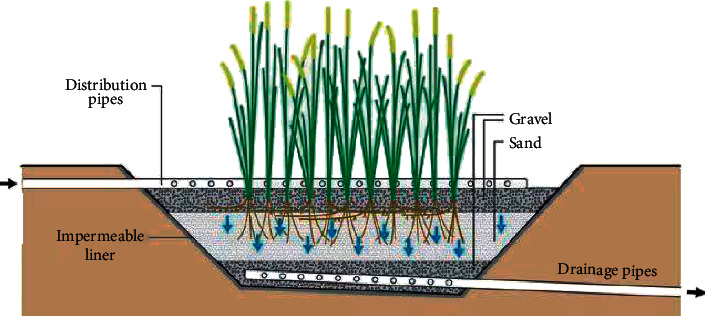
Schematic cross section of a vertical-flow constructed wetland (VFCW) (source: [13]).

The amounts of water in these structures are below the substratum floor. They are also complemented by a robust media root network of new power plants. In soil sand and gravel-based humid zones, CW with subsurface flow may be graded horizontally and vertically according to the direction of flow.  Figure 2 shows the schematic cross section of a horizontal-flow constructed wetland (HFCW), and Figure 3 shows the schematic cross section of a vertical-flow constructed wetland (VFCW). The substratum supports and attaches a surface for microorganisms that are able to reduce anaerobic (and/or anoxic, if nitrate is present) and organic CO_2_, CH_3_, and H_2_S pollutants. The substrate is also a simple filter to keep influential suspended solids and micro-organic solids, which then degrade themselves and become stable within the bed for long periods generally restricting the outflow of suspended solids. Subsurface systems are also referred to as plant filters, reed beds, root zone method, gravel-bed hydroponic filters, vegetated submerged beds, or artificial wetlands [13].

### 5.1. Horizontal Flow Systems (HFSs)

Wastewater is pumped into the inlet in horizontal flow systems and gradually flows into the porous environment below the surface of the bed on the horizontal path to the outlet zone where it is collected before exiting at the outlet by means of a control system level as shown in Figure 3. Wastewater will contact a network of aerobic, anoxic, and anaerobic areas during this transit. Roots and rhizomes that leak into the substrate are found in the aerobic areas [14, 15]. These systems require much greater vegetable beds so that phosphorous and nitrogen are removed effectively [16].

HFS is very efficient in removing organic matter and suspended solids. Rates of elimination of BOD range from 65% to 90%, and average effluent concentrations of BOD are below 30–70 mg/l. A typical TSS level for effluent is less than 10–40 mg/l and is 70–95% less. Pathogen removal amounts to 99 percent or more (2-3 log) of total coliforms [17]. Generally, tropical and subtropical climates hold the greatest potential for the use of HFSs. Cold climates tend to show problems with both icing and thawing. Water stress of plants in a HFS is an important issue to be considered, especially in households and lodge systems during periods without inflow (for example, during holidays and low season).

### 5.2. Vertical Flow Systems (VFSs)

VFSs are shallow excavations or above-ground constructions with an impermeable liner, either synthetic or clay, characterized by intermittent (discontinuous) loading and resting periods where wastewater percolates vertically through the substrate. Intermittent and batch loading enhance oxygen transfer and thus nitrification. The main purpose of plant presence in a VFS is to help maintain the hydraulic conductivity of the bed. Removal efficiencies in terms of BOD, COD, ammonia-N, and pathogens of the VFS are generally higher compared to the HFS. However, removal of suspended solids is somewhat lower than the HFS [18].

Average removal efficiencies are typically within a range of 75–95 percent and 65–85 percent in terms of BOD and TSS, respectively. Pathogen removal in terms of total coliforms is typically within a range of 2-3 log and can be as high as 5 log as seen in Nepal [19]. However, removal of total nitrogen is comparable with FWS and HF systems due to inability to provide denitrification. This could be resolved by recycling of the effluent into the pretreatment unit [20]. Removal of phosphorus is also comparable with other types of CW. However, one of the major threats of good performance of the VFS is clogging of the filtration substrate [21, 22]. Therefore, it is important to properly select the filtration material, hydraulic loading rate, and distribute water evenly across the bed surface in order to avoid overloading of certain parts of the surface.

Given their reliance on a well-functioning pressure distribution, they are more adapted to locations where natural gradients can be used, thus enabling the filter by gravity. Since flat areas require the use of pumps, they are thus dependent on reliable power supply and frequent maintenance [13]. VFS could be further categorized into downflow and upflow depending on whether wastewater is fed onto the surface or to the bottom of the wetland. VFS is primarily used to treat domestic or municipal sewage. The system has also been successfully applied to municipal, industrial (explosives, food processing, airport deicing water, and acid mine drainage), and agricultural (aquaculture and swine feedlot) wastewaters [23].

## 6. Hybrid Systems

**Figure 4:**
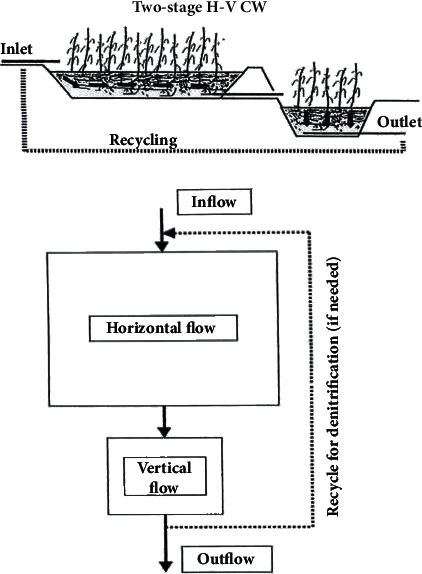
Schematic arrangement of the HF-VF hybrid system according to Brix and Johansen (source: [11]).

Various types of CW may be combined in order to achieve higher treatment effect, especially for nitrogen. In these systems, the advantages of the HSF and VF systems can be combined to complement each other, for instance, the case studies identified in Egypt. Therefore, there has been a growing interest in hybrid systems (also sometimes called combined systems). Hybrid systems comprise most frequently VFS and HFS arranged in a staged manner.  Figure 4 shows the schematic arrangement of the HF-VF hybrid system according to Brix and Johansen; however, all types of CW could be combined. It is possible to produce an effluent low in BOD, which is fully nitrified and partly denitrified and hence has much lower total-N concentrations [18, 24]. The design consists of two stages of several parallel VF beds (filtration beds) followed by 2 or 3 HF beds (elimination beds) in series. The results indicate very good removal for organics (BOD and COD) and TSS, while removal of nitrogen is enhanced with no nitrate increase at the outflow [24, 25].

Constructed wetland technology meets the basic criteria of sustainable sanitation systems by preventing diseases, protecting the environment, and being an affordable, acceptable, and simple technology. Additionally, CWs produce treated wastewater of high quality, which fosters reuse, which in turn makes them applicable in resource-oriented sanitation systems [7]. However, they are not recommended for treatment of raw wastewater. On the contrary, they have gained acceptance worldwide [26] due to their economically and environmentally sound attributes as a wastewater management option and as design, construction and operational experience have accumulated over the years.

## 7. Challenges Associated with CWs

The main disadvantage is the requirement of a large amount of space, which is always the case either in the combination of various techniques of horizontal and vertical subsurface flow or the use of other innovations. However, there is plenty of space available in the remote resorts and game lodges due to the low population density of these areas. In this respect, space limitation should not be a major hindrance or problem with regard to game lodges and resorts in the national parks and game reserves. Initial cost of CWs can be high and prohibitive, especially for small hospitality operations.

Geographical characteristics differ from place to place thereby presenting another challenge. This requires careful geological analysis of the site to evaluate the presence or absence of rocks and to evaluate solutions in order to minimize excavation costs. Oketch [27] identified poor understanding of CW potential as one of the challenges and constraints to overcome in adopting CWs as wastewater management systems in Kenya.

In temperate and high mountainous areas, altitude is a factor that must be assessed carefully, especially above 1400 m a.s.l.; this is because of issues such as access difficulty, sludge disposal difficulty, reduced energy availability, and problems linked to frost and seasonal closures. Another discussion is the plants that must be different according to the altitude: additionally an accurate and necessary study to use native and typical plants of that climate belt and region so as not to create alterations (for example, aquatic plants are generally quite invasive) due to the environment.

Access difficulty may also establish a factor in the choice of systems, privileging systems that need reduced maintenance and simple work that can be carried out directly onsite by the user and low or no waste material (as sludge) to have to take away. The more isolated the location is, the more problems could be linked to the continuous availability of electricity which should push to choosing systems that work by gravity without the aid of pumps or aeration systems, or in any case, with reduced consumption, even at lower altitudes. Seasonality such as peak and off-peak also poses a challenge because of the reduced and increased volume of wastewater through the system.

To summarize, this study explores the potential use and challenges of CWs for wastewater treatment and environmental conservation in game lodges and resorts in Kenya. Generally, the literature review highlights a number of benefits and potential uses, as well as challenges, associated with the adoption of CWs internationally. However, there is little documented research on CWs within the Kenyan national parks, game reserves and other wilderness areas.

## 8. The Study Methodology

The study adopted qualitative research methods including the literature review in the topic area, textual content analysis and thematic analysis to collect and analyze the data. The study relied on desktop research where in-depth review of the literature relating to waste management and wastewater management in hospitality and tourism industries was carried out. Authentic industry reports, peer-reviewed journals and internet articles and publications were reviewed. In this respect, the study was exploratory in nature relying mainly on secondary data.

## 9. Data Collection

Among others, the secondary sources of data included governmental reports, official reports by international agencies and private sector hospitality and tourism enterprise publications. Selected corporate reports and case studies as well as published peer-reviewed academic research on wastewater management and the use of wetland technology were reviewed. The focus was on identification of data related to the specific study objectives. Adopting a deductive approach, data were collected and analyzed qualitatively using the objectives of the study as the basis for searching and coding. Thematic analysis approach was used to identify the relevant themes emerging from the study. The study adopted a two-step strategy for data collection, analysis and interpretation. The first step involved reading, identifying and coding relevant information from the selected secondary sources based on the study objectives and questions. The second step involved comparing, synthesizing, and summarizing the findings across different sources in order to draw conclusions. Similar or related patterns were then combined to form broad themes.

## 10. Results and Findings

The following section presents the results according to the objective set for the study.

### 10.1. Objective One: Adoption of CWs by Hospitality Establishments

There are a number of case studies on the use of constructed wetlands as wastewater management systems in the international hospitality and tourism industries. The following are selected examples of successful projects as indicated in Table 1.

#### 10.1.1. Egypt's Two Treatment Schemes Consisting of an Upflow Anaerobic Sludge Blanket (UASB) Reactor Followed by Either Subsurface Flow (SSF) Or Free Surface Flow (FSF) Constructed

Upflow anaerobic sludge blanket (UASB) reactor was fed continuously with municipal wastewater through a connection from the sewerage system and fed into FWS and SSF wetlands. The results indicated an average value of 78% for COD and 78% for BOD reduction in the SSF constructed wetland, while for the FWS constructed wetland, the average removal values of COD and BOD were 68% and 78.5% [10]. Therefore, SSF constructed wetland as a posttreatment step after a UASB reactor is a promising technology for wastewater reclamation and reuse in arid and semiarid areas [10].

#### 10.1.2. Sharquiya Governorate, Northeast of Cairo, Egypt

Three-chamber septic tank (ST) of 56 m^3^ total volume was designed and manufactured from concrete followed by the subsurface flow (SSF) constructed wetland [28]. By subjecting raw wastewater to the ST, the removal rate for the TSS, BOD, and COD was 59%, 46%, and 41%, respectively, while the efficiency of the wetland reached 78% and 79% for the COD and BOD, respectively, while the overall removal efficiency of the combined treatment was 89% for the BOD and 87% for the COD [28].

#### 10.1.3. Combined Upflow Anaerobic Sludge Blanket (UASB) Reactor and Subsurface Flow (SSF) Constructed Wetland in Sakaka City

The results indicated that the efficiency of the UASB as a first treatment step for the removal of contaminants in wastewater was found to be 67.7% of COD, 71.4% of BOD, and 65.5% of TSS and as such the combination of UASB and SSF is an effective method for the treatment of sewage water in Sakaka city [9].

#### 10.1.4. Splash Wetland in Nairobi, Kenya

It is located in the southern portion of Nairobi and is covered with domestic sewage from two restaurants. The splash wetland is approximately 0.5 ha. It is a subsurface hydroponic system (GBH) that is supposed to contain three surface flow systems known as wetland cells [29]. This system consists of three surface-built systems.

#### 10.1.5. Hotel Relais Certosa in Florence

The system consists of an HF stage followed by a VF process, which provides secondary treatment of wastewater coming from the hotel. Up to 140 PE (28 m^3^/day) is the acceptable use. The bacterial tension is similar to the values for reuse [30].

#### 10.1.6. Castelluccio di Norcia by the Umbria Region in Italy

The network uses the FRB + VF scheme, and pretreatment systems are made up of an electronic panel. With a special self-priming syphon, massive volumes of water can easily be circulated throughout the first level, allowing optimal drainage delivery through the whole region of the basins. The network has a flood flow of about 2000 m^2^. Escape water slowly falls to the ground in an absorption state since no water supplies can be located on the plateau [30].

#### 10.1.7. Yucatan Peninsula, Southern Mexico

Starting in 1996, several dozen wastewater and subsurface-flowing wetlands were established in the south of Cancún, Mexico coastal Yucatan Peninsula, for houses, condominiums, restaurants, and small hotels. Experiments have begun with the use of plants with a high biodiversity, both native and precious in the area. Water was sent to subsurface drains to discharge the systems. The resulting trials showed a 65%–70% decrease in COD and a decrease in nutrient cuts [31].

#### 10.1.8. Resort Island of Bali, Indonesia

Most of the wastewater in Bali and Indonesia is completely untreated and floods to rivers as well as groundwater leading to an environmental damage. In Indonesia's tropical archipelago, the environment, material available and overwhelming needs for wastewater solutions can be used in a great environment for implementing the WWG technology [31].

### 10.2. Objective Two: Benefits, Potential Uses, and Challenges to Adopting CWs in the Hospitality Industry

Constructed wetlands are generally less expensive to build; characterized by simple construction, operation, and maintenance; have low operation and maintenance cost; high ability to withstand fluctuations in flow and inlet quality; high pathogen removal; good water reuse and recycling options; and optimal esthetic appearance [28]. Benefits from vegetation biomass in constructed wetlands are vital for provision of economic returns to communities when harvested for biogas production, animal feed, fiber for paper making and compost [28]. Studies conducted by the IHEI and Accor have shown 90% of hotel clients want to stay in an environmentally-friendly hotel (http:/www.hotelonline.com/News/3rd). Hotels have focused on environmental management practices in order to conserve energy, conserve water, reduce waste and develop good connections with local communities.

Where the quality of treated wastewater meets the set environmental standards, water could be used to create ponds for purposes of breeding and growing fish for consumption and replenishing the nearby rivers and water bodies, outdoor irrigation and watering flower gardens, animal drinking water points, flushing toilets, swimming pools, and landscaping. By doing so, the CWs will mitigate competition for the scarce resources such as water and fish, among others. Treated water could also be recycled for general cleaning and toilet flushing. In this regard, hospitality establishments adopting CW technology may benefit through cost reduction, increase in profits, and contribute to environmental conservation and sustainability. Game lodges and resorts adopting CWs may also benefit from increased competitiveness and improved corporate image, as well as higher customer retention by targeting environmentally conscious visitors.

#### 10.2.1. Objective Three: The Efficacy of Constructed Wetlands as Wastewater Management Systems in Remote Game Lodges and Resorts in Kenya

CWs have been envisaged as cost-effective wastewater treatment technologies suitable for developing countries [29, 32]. While initial costs may be high in the long run based on the cost benefit analysis, CWs are recommended as environmentally sound business investments. Additionally, the high levels of biodiversity in constructed wetlands permit multiple degradations implying higher performances compared to technological treatment plants that have little bacterial growth [33]. Also, there is no excess sludge to be removed as there are biomass growth and decomposition [34]. In general, CW technologies can be used to manage wastewater and reduce operational costs and environmental pollution within game lodges, ecolodges, and resorts in the wilderness areas, national parks, and reserves in Kenya. However, at present, CWs are not widely used in Kenya except in a few instances. This is usually on large scale mainly in municipalities, industries, hotels, or farms.

## 11. Conclusions and Recommendations

This study was undertaken to explore the potential use and challenges of constructed wetlands for wastewater treatment and environmental conservation in game lodges and resorts in hospitality and tourism industries in Kenya. The study uncovers that constructed wetlands are a viable environmentally-friendly natural wastewater management technology. The existing evidence suggests that constructed wetland technology can reduce contaminant load by an average of 75%, 86%, and 78.5% for total suspended solids (TSS), chemical oxygen demand (COD), and biochemical oxygen demand (BOD), respectively. Treated water from the CW could be recycled and used for a variety of purposes thereby resulting in reduction in operational costs, wastewater consumption, and environmental pollution, as well as increased profitability. These results have been summarized in Table 1. However, the study also reveals a number of challenges associated with the constructed wetland technology including accuracy of methods used. Based on the above, the following recommendations can be made.

### 11.1. Recommendations

1. There is a need to explore the use of CWs for wastewater management in the game lodges, ecolodges, and resorts located within the wilderness areas, national parks, games reserves, and forests.
2. With regard to wastewater management in national parks, game reserves, and other public outdoor areas, we recommend that policies and guidelines be formulated to ensure CWs in these areas promote conservation efforts without compromising the integrity of the local environment.
3. It is recommended that the hospitality and tourism establishments pretreat wastewater by filtering and using grease traps and other appropriate technologies to remove solid waste before discharging it into the CWs.
4. Due to the potential for overload, especially during rainy season, it is recommended that the design of any CW system integrates protective strategies to regulate the flow of floods into the systems.
5. There is a need to create awareness and educate hospitality managers on environmentally-friendly wastewater management systems including CWs as well as their associated socioeconomic benefits.
6. The SSF CWs are more suitable for game lodges and resorts in the tropical areas because of their effectiveness in removal of wastewater contaminants, they require a smaller area to build and there is no direct contact between the water column and the atmosphere thus reducing the problems associated with mosquitoes.

### 11.2. Future Research

This was a desk research and did not reveal any theoretical models to predict the degree or success levels of CWs in reducing the amount of contaminants to locally, environmentally acceptable standards. Such models could inform policy and managerial decision-making. This is an area requiring further research. Such research should focus on identifying and delineating the relevant factors and parameters that need to be considered in the development of the appropriate models in a given environment. It is suggested that pilot studies be conducted to determine the efficacy of CWs as wastewater management systems and potential challenges, as well as the attitudes and support for their use by game lodges, ecolodges, resorts, and tented camps.

## Figures and Tables

**Table 1:** Summary of CW case studies indicating specific reduction load levels.

Facility	Contaminant percentage reduction		Biochemical oxygen demand (BOD)/organic matter
	Total suspended solids (TSS)	Chemical oxygen demand (COD)	
Egypt		68%	78.5%
Sharquiya Governorate, Cairo, Egypt		87%	89%
Sakaka City	65.5%	67.7%	71.4%
Splash Restaurant, Nairobi	97.6	94.5	96.1
Hotel Relais Certosa, Florence	>90	>90	>90
Castelluccio di Norcia, Italy	—	98	—
Yucatan Peninsula Southern Mexico	44.4	—	87.9
Tourist Resort in Bali, Indonesia	69	64	40
Reduction average	**75**	**86**	78.5

Source, Author (2016).

## Data Availability

All data used for this study have been duly acknowledged within the study and are readily available.
